# Diosmin Alleviates Retinal Edema by Protecting the Blood-Retinal Barrier and Reducing Retinal Vascular Permeability during Ischemia/Reperfusion Injury

**DOI:** 10.1371/journal.pone.0061794

**Published:** 2013-04-24

**Authors:** Nianting Tong, Zhenzhen Zhang, Wei Zhang, Yating Qiu, Yuanyuan Gong, Lili Yin, Qinghua Qiu, Xingwei Wu

**Affiliations:** Department of Ophthalmology, Shanghai Jiaotong University Affiliated Shanghai First People’s Hospital, Shanghai, China; University of Cologne, Germany

## Abstract

**Background and Purpose:**

Retinal swelling, leading to irreversible visual impairment, is an important early complication in retinal ischemia/reperfusion (I/R) injury. Diosmin, a naturally occurring flavonoid glycoside, has been shown to have antioxidative and anti-inflammatory effects against I/R injury. The present study was performed to evaluate the retinal microvascular protective effect of diosmin in a model of I/R injury.

**Methods:**

Unilateral retinal I/R was induced by increasing intraocular pressure to 110 mm Hg for 60 min followed by reperfusion. Diosmin (100 mg/kg) or vehicle solution was administered intragastrically 30 min before the onset of ischemia and then daily after I/R injury until the animals were sacrificed. Rats were evaluated for retinal functional injury by electroretinogram (ERG) just before sacrifice. Retinas were harvested for HE staining, immunohistochemistry assay, ELISA, and western blotting analysis. Evans blue (EB) extravasation was determined to assess blood–retinal barrier (BRB) disruption and the structure of tight junctions (TJ) was examined by transmission electron microscopy.

**Results:**

Diosmin significantly ameliorated the reduction of b-wave, a-wave, and b/a ratio in ERG, alleviated retinal edema, protected the TJ structure, and reduced EB extravasation. All of these effects of diosmin were associated with increased zonular occluden-1 (ZO-1) and occludin protein expression and decreased VEGF/PEDF ratio.

**Conclusions:**

Maintenance of TJ integrity and reduced permeability of capillaries as well as improvements in retinal edema were observed with diosmin treatment, which may contribute to preservation of retinal function. This protective effect of diosmin may be at least partly attributed to its ability to regulate the VEGF/PEDF ratio.

## Introduction

Many ocular diseases, such as retinal vascular occlusion, acute glaucoma, diabetic retinopathy, and retinopathy of prematurity, are associated with retinal ischemia/reperfusion (I/R) injury [1]. In the retina, hypoxic–ischemic stress seems to be an important cause of blood–retinal barrier (BRB) breakdown [2]. The BRB plays an important role in the homeostatic regulation of the microenvironment and is essential for the normal structural and functional integrity of the retina. It consists of inner and outer components, the inner BRB (iBRB) being formed by the tight junctions between the neighbouring retinal capillary endothelial cells and the outer BRB (oBRB) by tight junctions between retinal pigment epithelial cells. As the BRB serves critical functions in the eye, the breakdown of BRB associated with increased vascular permeability, results in vasogenic edema and tissue damage, with consequent the serious vision loss.

The BRB protects the retina against molecules and pathogens circulating through the bloodstream [2]. This specialized neurovascular interface is functionally comprised of endothelial tight junctions (TJ). BRB leakage and the extravasation of blood constituents into the retina have been attributed in part to direct injury to the TJ structure. The TJ are important structural and functional components for maintenance of BRB integrity. The TJ-associated proteins zonular occluden-1 (ZO-1) and occludin [3] have been identified as TJ marker proteins. Changes in their distribution and expression have direct impacts on the state of the TJ, and thus influence BRB permeability. Previous studies confirmed that TJ integrity declines and paracellular permeability of BRB increases with reduced expression of the TJ-associated proteins ZO-1 and occludin, thus leading to vasogenic retinal edema secondary to retinal ischemia.

Diosmin, a naturally occurring flavonoid glycoside, was first isolated in 1925 from *Scrophularia nodosa*, and first introduced as a therapeutic agent in 1969. As a vascular-protecting agent, its mechanisms of action include improvement of venous tone, increased lymphatic drainage, protection of capillary bed microcirculation, inhibition of inflammatory reactions, and reduced capillary permeability [4]–[7]. This agent reduces the expression of endothelial adhesion molecules (ICAM1, VCAM1), and inhibits the adhesion, migration, and activation of leukocytes at the capillary level. This leads to a reduction in the release of inflammatory mediators, such as prostaglandin E2 (PGE2) and thromboxane A2 (TxA2) [8]. Furthermore, diosmin causes significant decreases in plasma levels of endothelial adhesion molecules and reduces neutrophil activation, thus providing protection against microcirculatory damage [9], [10].

In our previous study [11], we found a neuroprotective effect of diosmin against retinal I/R injury. Furthermore, as a naturally occurring flavonoid glycoside, diosmin is a vascular protective agent used to treat chronic venous insufficiency [12], hemorrhoids, lymphedema, and varicose veins. However, to our knowledge, there have been no previous studies of the protective effects of diosmin on retinal capillaries, preservation of the BRB, and reduction of retinal edema after retinal ischemia. Therefore, the present study was performed to test the hypothesis that diosmin could protect the retinal capillary system, reduce retinal edema, and prevent BRB disruption, and then to investigate the underlying mechanisms.

## Methods

### Ethics Statement

Animal experiments in this study were performed in accordance with the Association for Research in Vision and Ophthalmology (ARVO) Statement for the Use of Animals in Ophthalmic and Vision Research. All experiments were performed in accordance with protocols approved by the Ethics Committee of Shanghai First People's Hospital, Shanghai Jiaotong University, Shanghai, China. All surgeries were performed under sodium pentobarbital anesthesia, and all efforts were made to minimize suffering. Utimately, animals were sacrificed by excessive dosage of anesthesia.

### Animals

Healthy male Wistar rats each weighing 220–250 g were purchased from the Shanghai Laboratory Animal Center of the Chinese Academy of Sciences. The animals were housed under controlled conditions that included a 12-h light/dark cycle (08∶00–20∶00 light; 20∶00–08∶00 dark), temperature of 23–25°C, humidity in the range of 55%–60%, and with free access to standard food and drinking water.

### Transient Retinal Ischemia

All surgeries were performed under aseptic conditions. The rats were anesthetized by intraperitoneal (i.p.) injection of 1% pentobarbital sodium (10 mg/kg). Corneal analgesia was achieved using 1 or 2 drops of 0.4% oxybuprocaine hydrochloride, and pupillary dilatation was maintained with 0.5% tropicamide and 0.5% phenylephrine. Body temperature was maintained at 36.5–37°C using a heating blanket. After dilation of the pupil, the anterior chamber of the right eye was cannulated with a 30-gauge needle connected to a physiological saline reservoir. The intraocular pressure (IOP) was raised to 110 mmHg by keeping the reservoir at 150 cm above the eye, and retinal ischemia was confirmed by examination of the fundus. After 60 min, the IOP was returned to normal pressure by removing the infusion needle from the anterior chamber. Ofloxacin ophthalmic gel (0.3%) was applied topically to the eye before and after the procedure. Only the right eye was used in all experiments, and the anterior chamber of the left eye was similarly cannulated without raising the IOP; this represented an untreated control.

### Group Assignment and Drug Treatment

For intragastric administration, diosmin (D3525; Sigma- Aldrich, St. Louis, MO) was freshly prepared by diluting the powder in physiological saline. Rats were divided into two groups (n = 16/group): a physiological saline-treated group (Vehicle) and a 10 mg/kg diosmin-treated group (Diosmin). This dosage was chosen based on our previous study [11]. Animals were given physiological saline or diosmin intragastrically 30 min before the onset of ischemia, and then daily after I/R injury until sacrifice.

### Hematoxylin and Eosin Staining

At 24 h and 7 days postretinal I/R injury, the eyeballs were marked at the 12 o’clock position of the cornea with silk suture, and then enucleated and fixed in 4% paraformaldehyde at 4°C for 24 h. After fixation, the anterior segment was removed, and the posterior eyeball containing the optic disc was dehydrated in a graded ethanol series and embedded in paraffin. For hematoxylin and eosin (HE) staining, 5 µm thick sections were taken along the vertical meridian and observed under a light microscope (Leica, Heidelberg, Germany).

To quantify the edema and ischemic damage to the retina, we measured various layer thicknesses to quantify the degree of cell loss. The overall retinal thicknesses (OT, from the inner limiting membrane to the pigment epithelium), outer retinal thickness (ORT, from the outer plexiform layer to the pigment epithelium), and inner retinal thickness (IRT, from the inner limiting membrane to the inner nuclear layer) were measured. All measurements (400×) were made approximately 2–3 disc diameters from the optic disc. Three sections per eye were averaged.

Furthermore, in order to investigate the differences of the thickness between central and peripheral retina for the three groups, the overall thickness was counted at a 0.5-mm interval within 0.5–4.5 mm of the superior and inferior edges to the optic nerve head (ONH) by a research staff unaware of tissue identity.

### Electroretinography

Retinal neuronal functions were examined by electroretinography (ERG) (EP-1000; Tomey, Erlangen, Germany) 24 h and 7 days after I/R injury. After overnight dark adaptation, the rats were anesthetized under dim red illumination and their pupils were dilated with 0.5% tropicamide and 0.5% phenylephrine. The rats were placed in a stereotactic frame, lying on a heating blanket to maintain the body temperature at 37°C, and facing the stimulus at a distance of 20 cm. Stainless-steel wire (0.1 mm in diameter) loops were placed on the cornea to act as the corneal electrode after topical application of ofloxacin ophthalmic gel. Flash ERG responses were recorded from both eyes by corneal electrodes, with the reference electrode placed in the middle of the lower eyelid and the ground electrode near the tail. The responses to a light flash (2.5 cd s/m^2^) from a photic stimulator, Ganzfeld Q400 (Roland, Germany), were amplified, and the preamplifier bandwidth was set at 0.3–300 Hz. Full-field white-light stroboscopic flashes lasting 10 µs were presented at a rate of 1.0 per second. All recordings were taken in a darkened room under dim red illumination to ensure a completely dark-adapted state. The amplitude of the a-wave was measured from the baseline to the trough of the a-wave, while that of the b-wave was measured from the trough of the a-wave to the peak of the b-wave.

### Western Blotting

Retinas were isolated and sonicated in RIPA buffer (Beyotime Biotech, Jiangsu, China). Lysates were centrifuged at 16000 rpm for 5 min. Supernatant protein concentrations were determined by BCA protein assay (Beyotime Biotech). Aliquots of 50 µg of protein per sample were separated by SDS-PAGE and immunoblotted onto PVDF membranes. Membranes were blocked with 5% non-fat milk in 0.1% Tween-20 in Tris-buffered saline (TBST) for 1 h at 25°C. The membranes were then incubated with polyclonal rabbit anti-occludin (diluted 1∶500; Santa Cruz Biotechnology, Santa Cruz, CA), rabbit anti-ZO-1 (diluted 1∶500; Zymed, San Francisco, CA), or mouse anti-β-actin (diluted 1∶1,000; Beyotime Biotech) diluted in 1% BSA/TBST buffer overnight at 4°C, followed by the addition of horseradish peroxidase-conjugated secondary antibody (diluted 1∶1,000; Beyotime Biotech) for 1 h at room temperature. Immunoreactive bands were visualized by enhanced chemiluminescence (ECL) (Beyotime Biotech). The intensity of the bands was determined using densitometric analysis and normalized to that of β-actin. Western blotting analysis was repeated 2–3 times with 3–5 samples per group.

### Transmission Electron Microscopy

TJ ultrastructure was evaluated by transmission electron microscopy (TEM) [13]. Rats were deeply anesthetized by intraperitoneal (i.p.) injection of 1% pentobarbital sodium (10 mg/kg body weight). The heart was exposed and the left ventricles were perfused with 0.9% saline via a catheter through the artery until colorless infusion, followed by perfusion with a fixative consisting of 2.5% glutaraldehyde and 4% paraformaldehyde in 0.1 M phosphate-buffered saline (PBS, pH 7.4). The eyes were promptly enucleated and bisected at the equator, the lens was removed, and the posterior segments were prepared. After 6 h of fixation in 2.5% glutaraldehyde, the retinas were rinsed with PBS and postfixed in 1% OsO_4_ for 2 h at room temperature. The retinas were dehydrated in a graded alcohol series to 100% ethanol, then washed with 100% propylene oxide, and embedded in Spurr resin (Electron Microscopy Sciences, Fort Washington, PA). Ultrathin sections were doubled-stained with uranyl acetate and lead citrate and observed using a Hitachi H-500 transmission electron microscope (Hitachi Ltd., Tokyo, Japan).

### Blood–retinal Barrier Permeability

The BRB permeability was evaluated according to the method of Xu *et al*. [14], with some modifications. Briefly, approximately 24 h after I/R injury, the rats were anesthetized by intraperitoneal (i.p.) injection of 1% pentobarbital sodium (10 mg/kg body weight). The right jugular vein and right iliac artery were cannulated with 0.28- and 0.58-mm internal diameter polyethylene tubing filled with heparinized saline (400 units heparin/ml saline), respectively. Evans blue was injected through the jugular vein over 10 s at a dose of 45 mg/kg. Two minutes after the rats turned visibly blue, which confirmed their uptake and distribution of the dye, 0.1 ml of blood was drawn from the right iliac artery. Equal volumes of blood were then drawn at 15-min intervals up to 2 h after injection to obtain the time-averaged Evans blue plasma concentration. After the dye had circulated for 2 h, the chest cavity was opened, and rats were perfused via the left ventricle with 1% paraformaldehyde in 0.05 M citric acid (pH 3.5) at 37°C. Immediately after perfusion, both eyes were enucleated and bisected at the equator. The retinas were then carefully dissected away under an operating microscope. After measurement of the retinal wet weight, the Evans blue dye was extracted by incubating each retina in 0.3 ml of formamide (Sigma) for 18 h at 70°C. The extract was ultracentrifuged at 70000 rpm for 45 min at a temperature of 4°C to precipitate any proteins with absorbance at 620 nm. Aliquots of 60 µl of the supernatant were used for measurement of the absorbance at 620 nm. The concentration of dye in the extracts was calculated from a standard curve of Evans blue in formamide. BRB breakdown was calculated using the following equation, with the results expressed in µl plasma × g retinal wet wt^−1^·h^−1^.

BRB breakdown = (retinal Evan blue in micrograms/retina dry weight in grams)/(time-averaged plasma Evans blue in micrograms/plasma volume in microliters × circulation time in hours) and was expressed as microliters plasma/gram retina dry weight per hour.
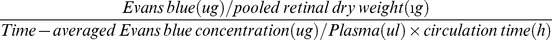



For qualitative observation of retinal capillary leakage, the rats were administered Evans blue (100 mg/kg) via the tail vein and kept on a warm pad for 2 h. The eyes were enucleated and fixed with 2% paraformaldehyde in PBS for 2 h. After 2 h, the retinas were dissected, and flat mounts were obtained and mounted on glass slides. Retinal flat mounts were visualized under a confocal microscope (Eclipse E800; Nikon, Tokyo, Japan).

### Enzyme-linked Immunosorbent Assay

VEGF and PEDF were measured in retinal tissue collected at 24 h and 7 d after reperfusion. For detection of VEGF and PEDF in retinal tissue, frozen tissue samples were weighed and homogenized. The homogenates were centrifuged at 120000 × *g* for 10 min and the supernatants were stored at–80°C prior to analysis. In each sample, the concentrations of VEGF (Rat VEGF Quantikine ELISA Kit; R&D Systems, Minneapolis, MN) and PEDF (Rat pigment epithelium-derived factor, PEDF ELISA Kit; EIAab*®,* Wuhan, China) were measured with ELISA kits according to the respective manufacturer’s protocol. Standard curves for each of the substances analyzed were included in each run, and sample concentrations were calculated.

### Immunohistochemistry Assay

Immunohistochemistry was performed as described previously [15]. Briefly, retinal sections were incubated with 1∶100 dilution of an anti-VEGF antibody (Santa Cruz Biotechnology, Inc., Santa Cruz, CA) and 1∶150 dilution of an anti-PEDF antibody (Santa Cruz Biotechnology, Inc., Santa Cruz, CA). After extensive washes, the sections were incubated with a biotin-labeled monoclonal anti-rabbit antibody and then developed using the ABC method (Vector Laboratories, Burligame, CA), with 3,3′ diaminobenzidine (0.025% in 0.05 M Tris, pH 7.4; containing 0.03% hydrogen peroxide) as a chromogen.

### Statistical Analysis

All of the results are expressed as the mean±standard deviation. Statistical analyses were performed using SPSS for Windows version 17.0 (SPSS, Inc., Chicago, IL). To identify significant differences, analysis of variance and the Student-Newman-Keuls test were applied. In all analyses, a value of *P*<0.05 was taken to indicate statistical significance.

## Results

### Improved Retinal Neuronal Function

**Figure 1:**
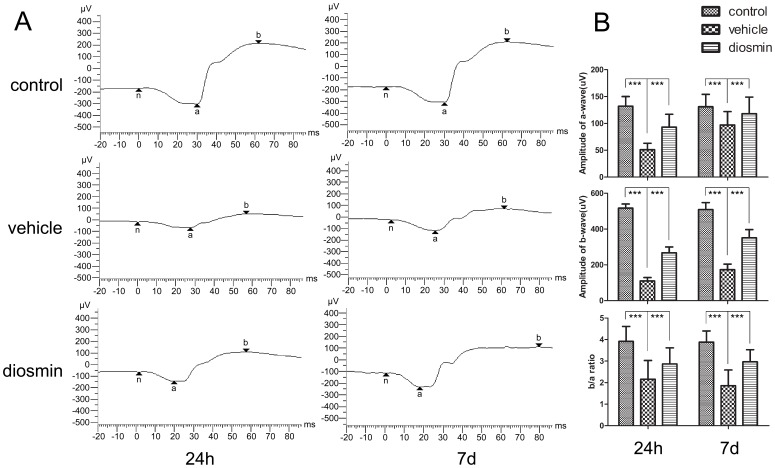
Effect of diosmin on the scotopic a- and b-wave amplitudes as assessed by ERG. (A) Typical ERG records for the three groups 24 hours and 7 days after ischemia. (B) The ERG was recorded for quantitative analysis. The a- and b-wave amplitudes were measured, and the b/a ratio was determined. Data are expressed as the mean±SD (*n* = 8). ***P<0.001.

ERGs were recorded in all experimental groups 24 h and 7 d after I/R injury (n = 8/group). Figure 1 A shows the typical ERGs for the three groups. Retinal ischemia led to significant decreases in the a-wave and b-wave amplitudes in the early stage. At 24 h after retinal ischemia, the a- and b-wave amplitudes in the Vehicle group were reduced by 61% and 79%, respectively, compared to those in the Control group (51.47±12.65 *vs*. 132.74±18.41 µV for a-wave and 110.12±19.21 *vs*. 517.77±23.55 µV for b-wave) (*P*<0.001, Figure 1 B) reflecting the retinal functional impairment in the early stage after I/R insult. The extent of amplitude reduction was much more significant in b-wave than a-wave, and this contributed to a remarkable decrease in the b/a ratio (Figure 1 B). At a later stage, 7 d after insult, there was a level of recovery in amplitudes of both a- and b-wave in the Vehicle group compared to the early stage in the same group. However, the a- and b-wave amplitudes in the Vehicle group were also reduced by 27% and 66%, respectively, compared with those in the Control group (97.01±21.84 *vs*. 131.96±23.72 µV for a-wave and 173.52±31.13 *vs*. 509.90±39.61 µV for b-wave) (*P*<0.001, Figure 1 B). Diosmin reversed the amplitude reduction induced by retinal ischemia at both 24 h (93.91±24.61 µV for a-wave and 267.31±33.57 µV for b-wave, *P*<0.001, compared with the Vehicle group) and 7 d (118.19±31.29 µV for a-wave and 351.02±46.61 µV for b-wave, *P*<0.001, compared with the Vehicle group) after retinal I/R injury (Figure 1 B).

### Decreased Retinal Edema

**Figure 2:**
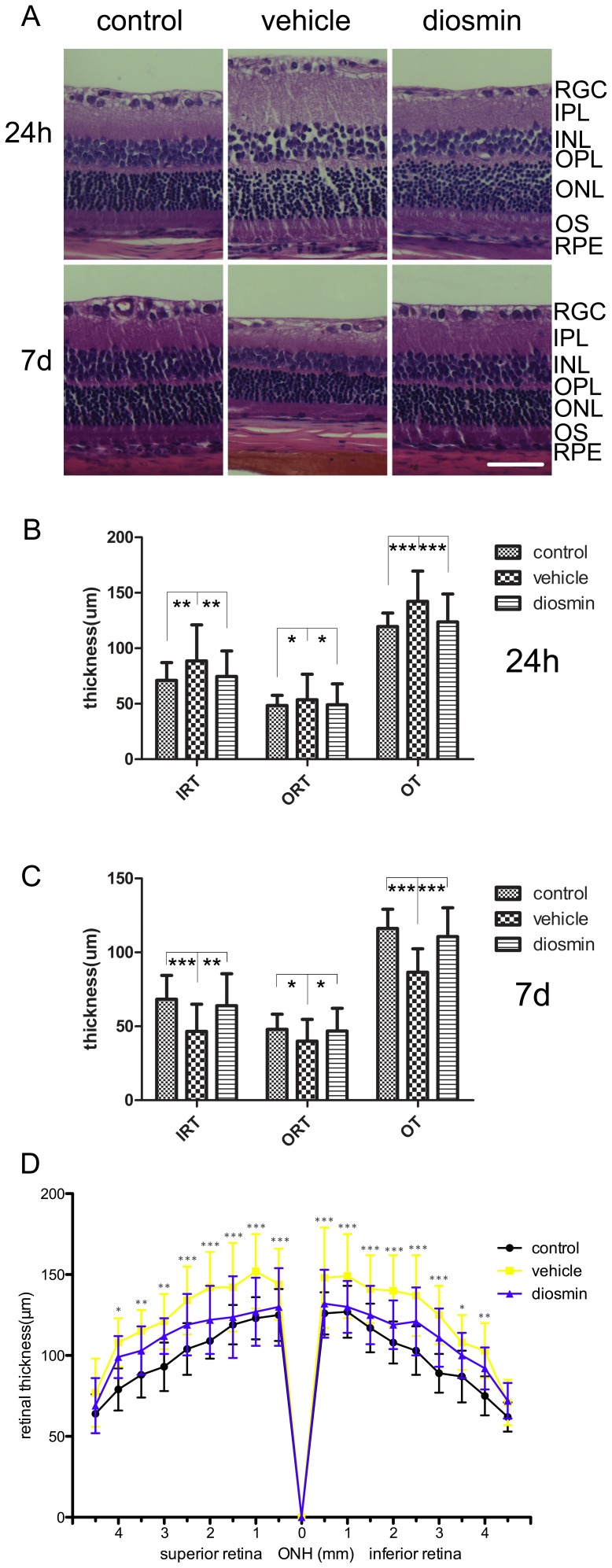
Histological examination after retinal ischemia. The retinal I/R injury induced retinal edema and retinal atrophy 24 h and 7 d after ischemia, respectively. These insults were reversed by diosmin, as shown in the HE analysis. (A) Representative photographs of rat retinas from the three groups 24 h and 7 d after ischemia. The thickness of the retinas was analyzed quantitatively (B) 24 h and (C) 7 d after ischemia. (D) Changes of the whole retinal thickness 24 h after retinal ischemia for three groups. RGC, retinal ganglion cells; IPL, inner plexiform layer; INL, inner nuclear layer; OPL, outer plexiform layer; ONL, outer nuclear layer; OS, outer segment; RPE, retinal pigment epithelium; IRT, inner retinal thickness; ORT, outer retinal thickness, OT, overall thickness, ONH, optic nerve head. Data are expressed as the mean±SD (*n* = 6). *P<0.05, **P<0.01, ***P<0.001. Scale bar, 50 µm.

I/R injury induced marked retinal swelling 24 h after insult (n = 6/group). There were significant increases in both inner retinal thickness (IRT) and outer retinal thickness (ORT), as well as overall retinal thickness (OT) in the Vehicle group as compared with the Control group (88.55±32.61 *vs*. 71.11±15.93 µm, *P* = 0.002; 53.64±22.98 *vs*. 48.41±9.17 µm, *P* = 0.023; 142.18±27.39 *vs*. 119.53±12.17 µm, *P*<0.001, respectively) (Figure 2 A, B). In contrast, there was a decrease in retinal thickness in the Diosmin group (74.52±23.04 µm for IRT, *P* = 0.003, 49.16±18.82 µm for ORT, *P* = 0.027, 123.69±25.15 µm for OT, *P*<0.001, respectively) as compared with the Vehicle group (Figure 2 A, B).

In consideration of the differences of the thickness between central and peripheral retina, we counted the retina at a 0.5-mm interval within 0.5–4.5 mm of the superior and inferior edges to the ONH, and found that except the retina which was 4.5 mm far from the ONH, the retinal thickness was significantly decreased in the diosmin group when comared with vehicle group, and the significant was more for cetral retinas than for peripheral retinas (Figure 2 D).

However, 7 d after retinal ischemia (n = 6/group), there were significant decreases in IRT, ORT, as well as OT in the Vehicle group as compared with the Control group (49.59±18.33 *vs*. 68.31±16.15 µm, *P*<0.001; 39.96±14.67 *vs*. 47.91±10.30 µm, *P* = 0.012; 86.54±15.89 *vs*. 116.23±13.01 µm, *P*<0.001, respectively) (Figure 2 A, C). Diosmin reversed this trend, and there was a significant recovery in retinal thickness (63.94±21.58 µm for IRT, *P* = 0.001, 46.75±15.42 µm for ORT, *P* = 0.015, 110.69±19.49 µm for OT, *P*<0.001, respectively) as compared with the Vehicle group (Figure 2 A, C).

### Preservation of Tight Junction Structure

**Figure 3:**
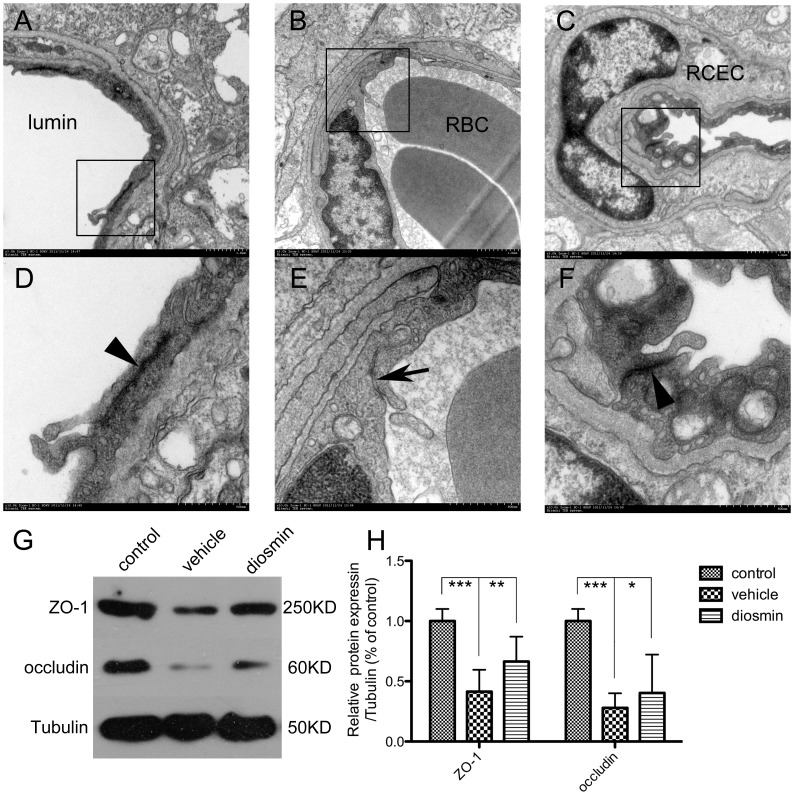
The ultrastructure of tight junctions (TJ) and the expression of TJ associated protein, ZO-1 and occludin. In control retinas (A, detail in D), the arrowhead shows “closed” tight junctions, which reflect the integrity of the TJ structure. Retinal ischemia induced “opened” tight junctions, as shown by the arrow in the vehicle groups (B, detail in E), indicating damage to the integrity of the TJ structure. Preserved “closed” tight junctions shown by the arrowhead after diosmin treatment (C, detail in F) indicated its protective effect on maintaining the integrity of the TJ structure after ischemic insult. A-C, magnification × 45,000; D-F, magnification × 150,000. (G) Representative Western blot showing the expression of ZO-1 and occludin. (H) Quantitative analysis of the Western blot. RBC, red blood cells; RCEC, retinal capillary endothelial cells. Data are expressed as the mean±SD (*n* = 6). *P<0.05, **P<0.01, ***P<0.001.

24 h after retinal I/R injury, electron microscopic examination revealed “closed” TJ in the Control group, (Figure 3 A, D). In contrast, “opened” TJ were found in the Vehicle group (Figure 3 B, E). Diosmin preserved the integrity of the TJ structure and they were “closed” in the Diosmin group (Figure 3 C, F).

Furthermore, as the TJ marker proteins ZO-1 and occludin are pivotal to maintenance of the integrity of the BRB, we explored the expression of these proteins by western blotting to investigate the effects of diosmin in preserving the structure of TJ (n = 6/group). Figure 3 G shows representative graphs for the three groups. As shown in Figure 3H, the expression levels of these proteins in the Vehicle group were significantly decreased 24 h after I/R injury compared with the Control group (*P*<0.001 for both proteins). Diosmin significantly reversed this trend, and the expression levels of ZO-1 and occludin were markedly higher than those in the Vehicle group (*P* = 0.004 and *P* = 0.038 respectively).

### Alleviated Blood–retinal Barrier Breakdown

**Figure 4:**
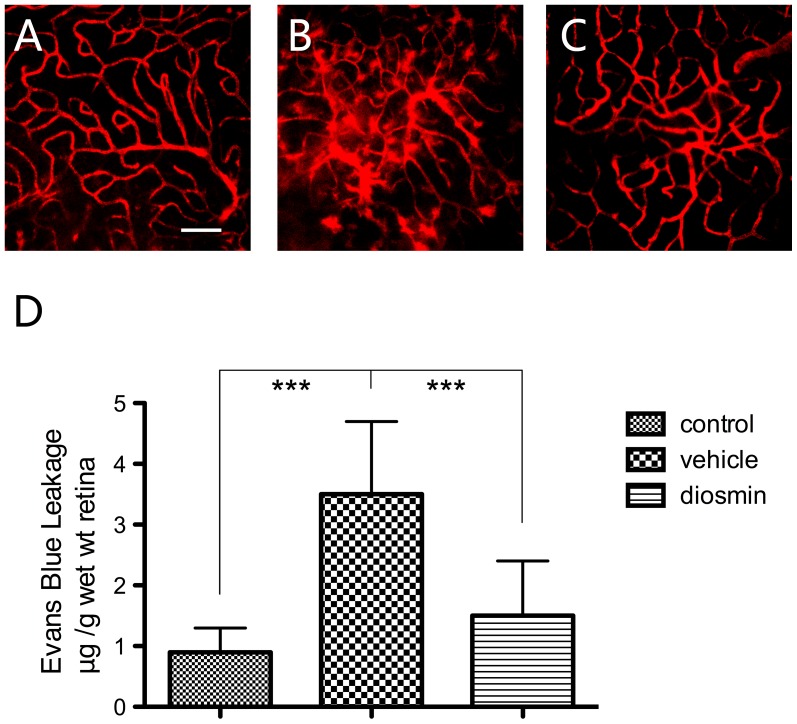
Visualization of retinal blood vessel leakage after intravascular perfusion with Evans Blue. (A) Low levels of background fluorescence and vasculature sharply outlined by the dye are seen in control retinas. (B) Twenty-four hours after ischemia, focal sites of leakage and diffusely distributed dye are found in the vehicle retinas. (C) Diosmin treatment markedly reduced the vascular permeability. (D) Quantitative analysis of the Evans Blue leakage. The photographs are representative of four eyes per group and the data are expressed as the mean±SD (*n* = 6). ***P<0.001. Scale bar = 100 µm.

I/R injury increased the BRB permeability in the Vehicle group in comparison with the Control group (3.5±0.7 *vs*. 0.9±0.4 µl·g^−1^·h^−1^, *P*<0.001) 24 h after insult. In the Diosmin group, there was a significant decrease in BRB permeability (1.5±0.9 µl·g^−1^·h^−1^) compared with Vehicle group (*P*<0.001) (Figure 4D). Blood vessel leakage was also visualized with Evans blue in retina flat mounts. In Untreated Control retinas, Evans blue fluorescence was limited to the blood vessels, whereas in the Vehicle group, focal leakage of the dye from capillaries and larger vessels was detected, and treatment with diosmin prevented this effect (Figure 4A–C), corroborating the data obtained with the quantitative Evans blue assay.

### Regulation of the VEGF/PEDF Ratio

**Figure 5:**
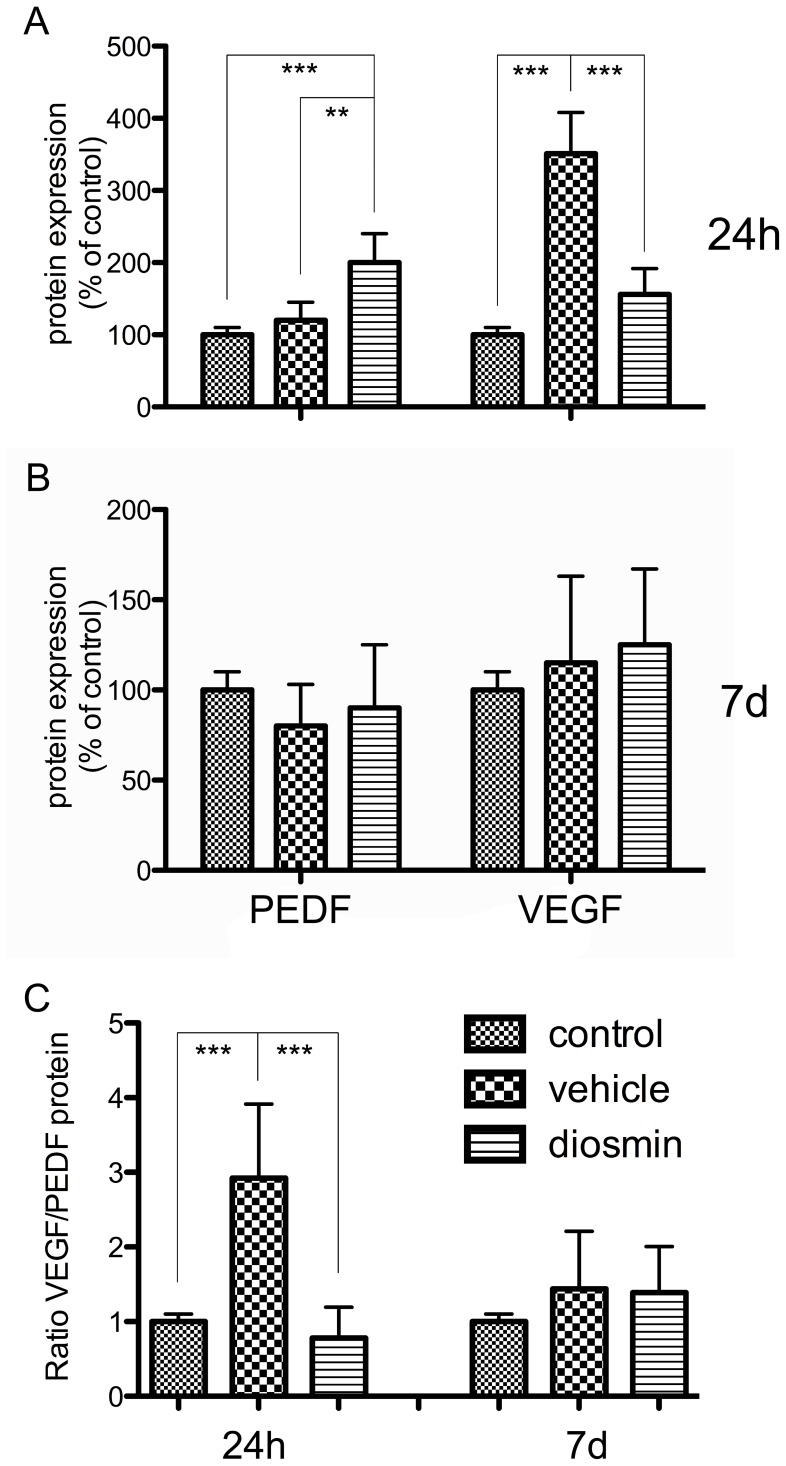
ELISA analysis of VEGF and PEDF protein expression. (A) Twenty-four hours after ischemia, there was a significant increase in VEGF expression and a slight increase in PEDF expression in the vehicle group compared with the control group; diosmin treatment reversed this trend. (B) Seven days after ischemia, the VEGF and PEDF protein expression returned to normal in both the vehicle and diosmin groups. (C) The VEGF/PEDF ratio for the three groups 24 h and 7 d after ischemia. Data are expressed as the mean±SD (*n* = 8). **P<0.01, ***P<0.001.

At 24 h after I/R injury, the levels of VEGF and PEDF expression were increased to 351% (*P*<0.001) and 120% (*P* = 0.057), respectively, in the Vehicle group as compared with the Control group (Figure 5A). This significant increase in the expression of VEGF and slight increase in that of PEDF resulted in a marked increase in the VEGF/PEDF ratio in the Vehicle group as compared with Control group (Figure 5C). Diosmin reversed this tendency. As shown in Figure 5A, in the Diosmin group, the level of VEGF expression was decreased by 56% (*P*<0.001) and that of PEDF was increased by 67% (*P* = 0.004) in comparison with the Vehicle group. As shown in Figure 5C, there was a corresponding decrease in the VEGF/PEDF ratio in the Diosmin group in comparison with the Vehicle group (*P*<0.001).

**Figure 6:**
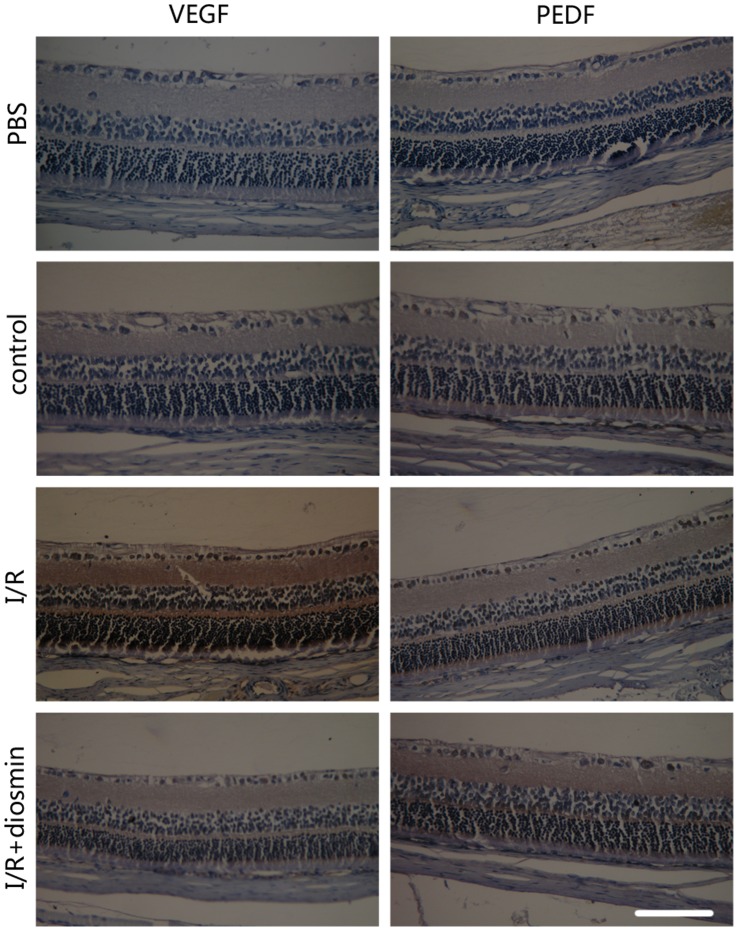
The Immunoreactivity for VEGF and PEDF in retina. No significant immunoreactivity for VEGF and PEDF was found in control retina. I/R injury induced a stronger immunoreactivity for VEGF in retina and diosmin administration relieved this trend. The PEDF immunoreactivity was nearly similar to the control group after 24 h retinal ischemia, while diosmin administration immensely increased this immunoreactivity. Scale bar, 100 µm.

Localization of PEDF and VEGF protein in the retina was shown in Figure 6. Immunoreactivity for VEGF and PEDF was mainly detected in the inner part of the retina. Consistant with the result of ELISA, ischemic insult resulted in a significant stronger immunoreactivity for VEGF and similar immunoreactivity for PEDF when compared with the control retina. Diosmin administration immensely reversed the trend for VEGF and greatly incresed the immunoreactivity for PEDF.

However, at 7 d after I/R injury, the levels of VEGF and PEDF expression returned to normal and the differences were not significant among the three groups (*P*>0.05) (Figure 5B), and accordingly, the VEGF/PEDF ratio decreased to similar to be similar to control group (*P*>0.05) (Figure 5C).

## Discussion

A number of *in vivo* mammalian models of retinal ischemia have been developed. High IOP-induced ocular ischemia is a frequently used model and has been described in a number of species. This method produces global ischemia, with obstruction of both the retinal and uveal circulation, as evidenced by flattening of the ERG, whitening of the fundus, and iris pallor. Although there are important differences between retinal blood supply in humans and rats, of the readily available laboratory animals, the rat is probably the optimal and most widely used animal for retinal ischemia research [1]. Therefore, in the present study, unilateral rat retinal ischemia was induced by elevating the IOP above the systolic blood pressure for 60 min, followed by a drastically reduced blood supply to the retina, as indicated by whitening of the fundus.

The BRB plays an important role in maintaining the homeostatic condition of the retinal microenvironment and excluding harmful substances. Retinal ischemia occurring in many clinical ischemic conditions, including retinal vascular (artery and vein) occlusion, acute glaucoma, diabetic retinopathy, and retinopathy of prematurity, results in breakdown of the BRB. Disruption of the BRB associated with increased vascular permeability results in vasogenic edema and tissue damage, with consequent adverse effects upon vision.

In the present study, we investigated the expression of the TJ marker proteins, ZO-1 and occludin. In accordance with the changes in TJ observed by TEM, retinal IR injury significantly reduced the levels of ZO-1 and occludin expression and resulted in “opened” TJ. The decreased expression levels of these proteins were attributed to the increased expression of VEGF under ischemic stimulus. VEGF, which was originally referred to as vascular permeability factor (VPF), is a potent microvascular permeability-enhancing cytokine [16]. Increased expression of VEGF has been reported in the ischemic retina and inhibition of VEGF production has been reported to reduce BRB permeability [17]. Müller cells and astrocytes were identified as the main source of VEGF expression [18]. In the brain, upregulation of endogenous VEGF in ischemia is believed to interact with receptors for VEGF on ischemic vessels and to contribute to disruption and leakage of the blood–brain barrier (BBB) [19]. VEGF binds to and activates two tyrosine kinase receptors, VEGFR1 and VEGFR2. The activation of these receptors is known to contribute to disruption of TJ by decreasing occludin expression and may be important in the pathogenesis of BRB dysfunction [20]. Another study reported that hypoxia increases the paracellular flux across the cell monolayer via the release of VEGF, which in turn leads to decreased expression and enhanced phosphorylation of ZO-1 [21]. In addition to these factors, VEGF may also increase the retinal vascular permeability by inducing inflammatory changes in the retina. ICAM-1 is a pivotal factor in these effects. Previous studies have confirmed that VEGF is one of the most important mediators involved in upregulation of ICAM-1 expression in endothelial cells both *in vivo* and *in vitro* [22], [23]. Retinal leukocyte adhesion to the vascular endothelium and leukostasis increase followed by increased expression of ICAM-1, and then capillary occlusion and endothelial cell apoptosis resulting in BRB breakdown are induced by leukocytes that are adherent to the vascular endothelium [24]. Furthermore, the previous observation that ICAM-1 inhibition significantly reduces BRB permeability represents further evidence for its role.

As VEGF is a potent factor involved in the induction of retinal permeability under conditions of ischemia, there should be antipermeability factors to resist the effect of VEGF in tissue. PEDF is a 50-kDa non-inhibitory member of the serine protease inhibitor (Serpin) gene family, and a potent antiangiogenic factor [25]. PEDF is secreted by many retinal cells, including Müller cells, vascular endothelial cells, pericytes, and retinal pigment epithelial cells, and has been reported to inhibit neovascularization in animal models for diabetes, prevent the accumulation of advanced glycation end products, reduce oxidative damage, and reduce inflammation. Numerous studies have demonstrated that PEDF inhibits VEGF-induced vascular permeability both *in vitro* and *in vivo* [26]–[28]. Furthermore, since VEGF and PEDF are regarded to act as a pair of factors, their ratio has been used as an indicator in many clinical and scientific studies[29]–[32]. Therefore, in the present study, the levels of VEGF and PEDF expression were determined and the VEGF/PEDF ratio was investigated. The increasing level of protein expression under conditions of ischemic stress was significant for VEGF but slight for PEDF, and the ratio of VEGF/PEDF was significantly decreased.

Our data indicated that diosmin has protective effects on the integrity of the BRB under ischemic conditions. At 24 h after retinal IR injury, obvious retinal edema was found, as well as the corresponding retinal functional injury, damage to the structure of TJ, loss of BRB integrity, and obvious retinal microvascular hyperpermeability. Diosmin treatment significantly reversed the effects of this insult, as indicated by recovered retinal function, preserved TJ structure, maintenance of the BRB integrity, and reduced retinal permeability accompanied by decreased retinal edema.

Retinal edema is an early complication secondary to retinal ischemia. Increased permeability of the blood vessels in the ischemic retina was detected in the present study. Leakage of intravenously administered tracers, such as Evans Blue, from the blood vessels into the various layers of the retina following IR injury in rats indicated that the integrity of the BRB was compromised. Although the retinal edema declined 7 d after the insult, VEGF expression reached levels not significantly different from those of the control group and the VEGF/PEDF ratio tended to normalize accordingly, the retinal function was significantly reduced and did not recover. These results indicate that retinal edema should be taken seriously and confirmed the importance of early intervention for retinal ischemia.

In conclusion, our data indicated that diosmin is effective in protecting TJ, maintaining the BRB integrity, and reducing retinal vascular permeability during retinal IR injury. This protective effect of diosmin seems to be at least partly due to regulation of the VEGF/PEDF ratio. Diosmin is thus a potential vascular protective drug for retinal edema associated with IR injury.
